# Facilitating hand hygiene in displacement camps during the COVID-19 pandemic: a qualitative assessment of a novel handwashing stand and hygiene promotion package

**DOI:** 10.1186/s13031-022-00492-8

**Published:** 2022-12-16

**Authors:** Sian White, Anika Jain, Abie Bangura, Michelle Farrington, Melaku Mekonen, Bang Chuol Nhial, Enamul Hoque, Md. Moniruzzaman, Pascaline Namegabe, John Walassa, Fiona Majorin

**Affiliations:** 1 Independent Consultant, New York, USA; 2 Department of Disease Control, London School of Hygiene and Tropical Medicine, Keppel Street, London, UK; 3 Oxfam, John Smith Drive, Oxford, UK; 4 Oxfam in Ethiopia, Bole Sub-city, Addis Ababa, Ethiopia; 5 Department of Public Health, Gambella University, Gambella Town, Gambella, Ethiopia; 6 Oxfam in Bangladesh, RAOWA Complex, VIP Road, Dhaka, 1206 Bangladesh; 7 Oxfam in DRC, Goma, Democratic Republic of Congo

**Keywords:** Handwashing, Displacement camps, Refugees, Hygiene promotion, COVID-19

## Abstract

**Background:**

Handwashing with soap is critical for the prevention of diarrhoeal diseases and outbreak related diseases, including interrupting the transmission of COVID-19. People living in large displacement settings are particularly vulnerable to such outbreaks, however, practicing handwashing is typically challenging in these contexts.

**Methods:**

We conducted a qualitative assessment of the implementation of a combined intervention to facilitate handwashing behaviour in displacement camps and in surrounding communities in Bangladesh, Ethiopia and the Democratic Republic of Congo during the COVID-19 pandemic. The intervention comprised a ‘hardware’ infrastructural component (provision of the Oxfam Handwashing Station) and a ‘software’ hygiene promotion package (Mum’s Magic Hands). We used programmatic logbooks, interviews with implementation staff and focus group discussions with crisis-affected populations to assess the use, feasibility and acceptability of the intervention.

**Results:**

Both components of the intervention were viewed as novel and appealing by implementing staff and crisis-affected populations across the study sites. The acceptability of the handwashing station could be improved by redesigning the tap and legs, exploring local supply chain options, and by providing a greater number of facilities. The implementation of the hygiene promotion package varied substantially by country making it challenging to evaluate and compare. A greater focus on community engagement could address misconceptions, barriers related to the intuitiveness of the handwashing station design, and willingness to participate in the hygiene promotion component.

**Conclusions:**

The combination of a ‘hardware’ and ‘software’ intervention in these settings appeared to facilitate both access and use of handwashing facilities. The acceptability of the combined intervention was partially because a great deal of effort had been put into their design. However, even when delivering well-designed interventions, there are many contextual aspects that need to be considered, as well as unintended consequences which can affect the acceptability of an intervention.

**Supplementary Information:**

The online version contains supplementary material available at 10.1186/s13031-022-00492-8.

## Introduction

The COVID-19 pandemic has emphasised the importance of hand hygiene for interrupting transmission of SARS-COV-2 [1, 2] and reemphasised its broader role in addressing long-term communicable disease challenges such as diarrhoeal disease [3] and other respiratory infections [4–6]. Prior to the pandemic, handwashing rates were low globally, with one study estimating that, on average, 26% of faecal contacts are followed by handwashing with soap [7]. Fear associated with the pandemic appears to have resulted in short-term increases in handwashing behaviour in many countries [8, 9]. However, rates of self-reported handwashing behaviour continued to change over the course of the pandemic in response to emerging evidence and changes in transmission, guidelines, national priorities, and risk perceptions [10, 11]. Given that handwashing is a socially desirable behaviour, actual practice is likely to be lower than self-reported estimates [12] and global surveys indicate that handwashing rates remained much less frequent in low- and middle-income countries (LMICs) [10, 13].

A major barrier to hand hygiene in LMIC settings is access to handwashing facilities with soap and water present. The availability of handwashing facilities is understood to be one of the strongest determinants of handwashing behaviour [7, 14] as it has the potential to both cue behaviour at critical moments and make the behaviour more convenient to practice. However, 26% of the global population lacks access to handwashing facilities with available soap and water, with this rising to more than 50% in the poorest regions of the world [15]. Furthermore, 70% of people in LMICs experience water scarcity issues that may create barriers for the adoption of COVID-19 prevention behaviours like handwashing [16]. Within refugee camps and displacement settings, handwashing facilities are often inadequate in number and in a state of disrepair, while soap and water are scarce and commonly prioritised for other tasks (e.g. laundry, bathing, cooking) [17–20]. Given these inadequacies in hygiene access and the densely populated living environments within displacement camps, crisis-affected populations were considered to be particularly vulnerable to COVID-19 [21].

In response to the challenges posed by the pandemic in displacement settings, Oxfam scaled up two hygiene promotion interventions that were designed for use in humanitarian crises. This included the procurement and installation of their Oxfam Handwashing Station (OHS) and a complementary hygiene promotion package called Mum’s Magic Hands (MMH). This qualitative study is designed to assess the feasibility, acceptability and experiences of use of these interventions in Bangladesh, Ethiopia and the Democratic Republic of the Congo (DRC) during the pandemic.

## Methods

A mixture of methods were used to understand the process of intervention implementation by Oxfam staff, the feasibility and acceptability of the intervention, and the exposure and use of the intervention by crisis-affected populations across three countries. Table 1 presents a summary of the methods used.

**Table 1:** Summary of methods

Method	Details	Objectives
Intervention logs	Standardised forms completed by Oxfam staff that covered information on shipment, delivery of the OHS, training provided to staff and community volunteers, soap distributions, hygiene promotion activities and maintenance of the OHS	To document intervention implementation
In-depth interviews with implementation staff	Interviews with Public Health Engineers and Public Health Promotors within Oxfam in each country	To get feedback from technical staff on their experiences of deploying OHS and MMH and feasibility of the intervention
Focus group discussions with crisis-affected populations	8 FGDs per country: 2 with adult men, 2 with adult women, 1 with older men (over 60), 1 with older women (over 60), 1 with younger people (18–25) and 1 with people with physical disabilities	To gain feedback from recipients of the intervention on access, acceptability, satisfaction, ease of use and maintenance of the OHS and MMH

### Study sites

The three study countries were purposively selected based on Oxfam’s COVID-19 programming in humanitarian locations. Within these countries, individual camps were selected according to identified gaps in hygiene programming and access to handwashing facilities. In Bangladesh, the intervention was implemented by Oxfam in three camps for Rohingya refugees in Cox’s Bazar District (Camps 3 and 4 in Ukhiya Upazilla and Camp 22 in Teknaf Upazilla. It was also implemented by other WASH sector partners in neighbouring host communities. In Ethiopia, the intervention was implemented by Oxfam in Nguenyiel Refugee Camp for South Sudanese refugees in Gambella Region and in Kebribeyah Refugee Camp. In the Democratic Republic of the Congo (DRC), the intervention was implemented in camps for internally displaced persons (IDPs) who were displaced by regional conflict in Tanganyika province (Kisabala and Kikumbe camps). In all sites water, sanitation and hygiene (WASH) services were provided by government or humanitarian actors and populations had been exposed to some hygiene programming prior to and during the pandemic. Basic handwashing facilities such as buckets with taps or ‘tippy taps’ did exist in all of the camps prior to the intervention, but there were not enough facilities to cater for the size of the populations and many were dysfunctional. Additional file 1 provides images of some of the existing handwashing facilities. Given that the intervention took place during COVID-19 there were also a range of other actors working in these camps who were promoting hygiene as part of COVID-19 prevention.

### Description of the intervention

The intervention, which aimed to increase handwashing rates among people living in displacement camps, consisted of a ‘hardware’ component—the OHS—and a ‘software’ component—MMH. Prior to the pandemic, Oxfam had worked with partners to develop the OHS and MMH approaches. Both the OHS and MMH have been continuously developed based on behavioural theory and iterative research and piloting in humanitarian settings [22, 23].

**Fig. 1:**
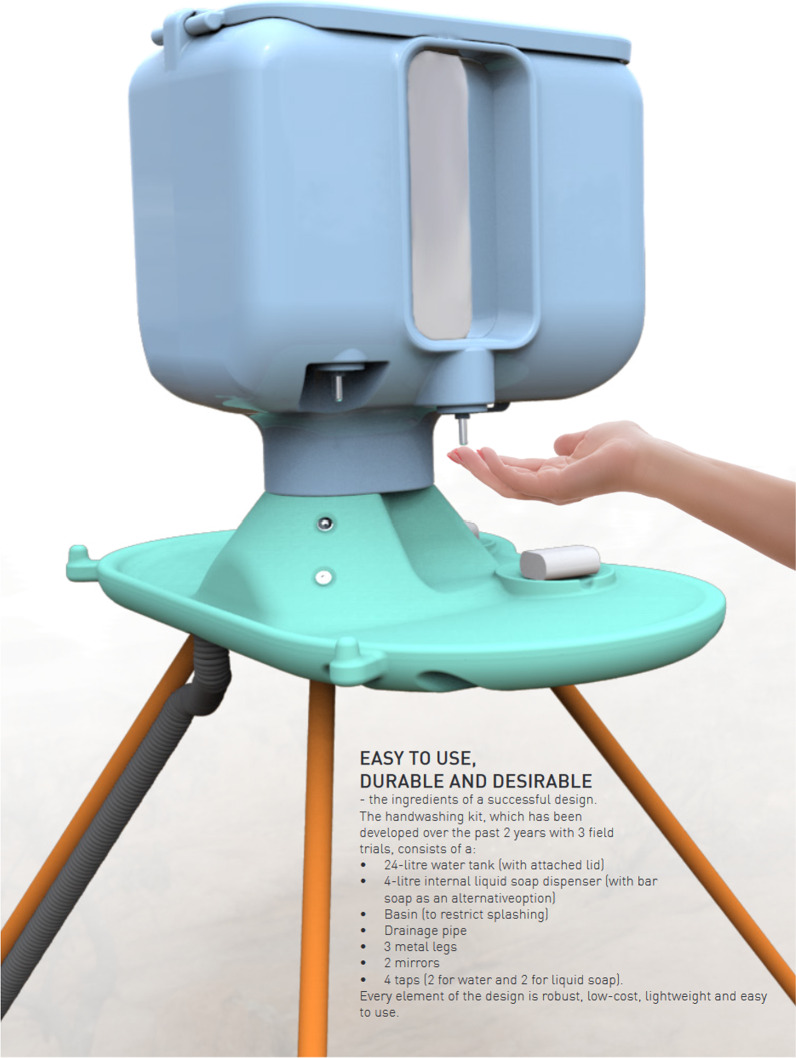
The Oxfam Handwashing Stand and a summary of its key features

The OHS is pictured in Fig. 1 with a description of key features. It is a handwashing facility that is designed to be desirable and easy to use, water-saving, durable, low-cost, and easy to transport and install. As part of COVID-19 response 1000 OHSs were procured and installed in camps and host communities in Bangladesh, 500 in the camps in Ethiopia and 500 in the camps in DRC. These were installed primarily in public or shared spaces such as at communal latrines, within shared compounds or at markets, schools or health facilities. Oxfam also set up WASH Committees, user groups and Community Health Volunteers to support the ongoing maintenance of the facilities.

**Fig. 2:**
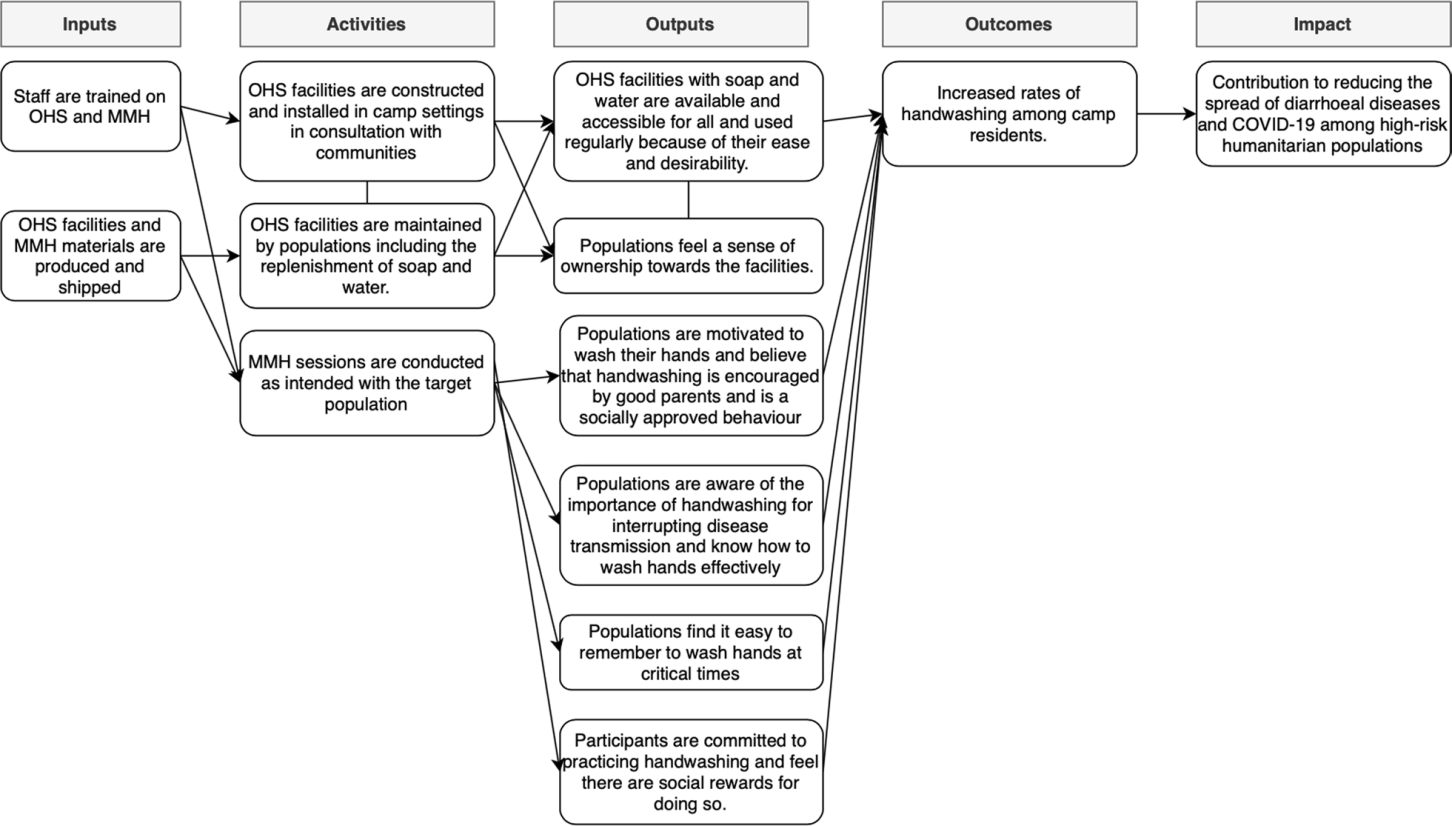
Intended theory of change for the combined delivery of the Oxfam Handwashing Stand and Mum's Magic Hands

To support increased use of the OHS, staff were also trained on MMH. MMH is a set of linked hygiene promotion activities and materials that can be context adapted. Overall the MMH activities are designed to change behaviour through four mechanisms: (1) to motivate hand hygiene by associating it with the motives of nurture (the desire to do what is best for your child) and affiliation (the desire to fit in with a social group); (2) to increase awareness about effective handwashing practice; (3) to reinforce behaviour at critical times through cues and reminders; and (4) to encourage participants to commit to practicing regular handwashing and reward them for doing so. A description of the intervention components for MMH is provided in Additional file 2 with an explanation of how each activity is designed to influence behaviour. This table also explains which components of MMH were selected to be implemented in each country. The MMH intervention was targeted at caregivers (including mothers, fathers, and grandparents) and children. The implementation of both MMH and OHS took place in June and July 2021 in all settings. Figure 2 describes the intended theory of change for both intervention components in order to contribute to an increase in hand hygiene behaviour in these humanitarian settings.

### Programmatic reporting via Intervention logs

The logs were designed to be a way of capturing key aspects of the implementation of the OHS and MMH. These were standardised Microsoft Excel templates that were completed by Oxfam staff and covered information related to the shipment and delivery of the OHS, training provided to staff and community volunteers, soap distributions, hygiene promotion activities and maintenance of the OHS.

### In-depth interviews (IDIs)

IDIs took place 3–4 months after the OHS and MHM implementation (September and October 2021). They were conducted with Public Health Engineers and Public Health Promotors within Oxfam in each country. IDIs followed an in-depth interview guide and were designed to assess the feasibility of delivering the OHS and MMH interventions, compare implementation experiences to the prior experiences that staff may have had with other hygiene promotion approaches, identify strengths and challenges with the intervention, and get their opinion on community reactions to the intervention. A list of all staff involved in implementation was developed and used as a sampling frame to purposively select staff to ensure a mix of roles, experience, and gender. IDIs were typically 30–45 min in duration and conducted in French, English or Bangla. IDIs were audio recorded, transcribed and translated to English as necessary.

### Focus group discussions (FGDs)

FGDs happened concurrently with the IDIs. In each country 8 FGDs were done with crisis-affected populations residing in the implementation areas. An FGD guide was designed to explore familiarity with the OHS, perceptions of it in relation to other common handwashing stations in the camps, and personal experiences of OHS use, maintenance and ownership. FGDs were segregated based on certain key characteristics so that in each country 2 took place with adult men, 2 with adult women, 1 with older men (over 60), 1 with older women (over 60), 1 with younger people (18–25) and 1 with people with physical disabilities. Data collectors worked with volunteer hygiene monitoring staff to select participants. They recruited participants at OHS facilities to ensure that all participants had used the facility at least once. Recruitment of older people and people with disabilities was done by working with camp management, community leaders and implementation staff in cases where there were pre-existing lists of people in these categories. If such lists were unavailable, staff went house to house in regions where the OHS were installed and selected participants by asking their age or using the Washington Group short set of questions for assessing functional limitations [24]. Due to COVID-19, FGDs were limited to a maximum of 7 people from neighbouring households (people who are likely to interact on a regular basis anyway). All data collectors and participants washed their hands before and after the FGD, wore masks throughout and were physically distanced. FGD locations were selected to be close to households (to minimise the need to travel) and be outdoor or in an area with good ventilation. FGDs were conducted in local languages, (Ruáingga, Nuer, Congolese Swahili) and were an hour to an hour and a half in duration. FGDs were audio recorded, transcribed and translated to English.

### Data collection and analysis

IDIs and FGDs were conducted and overseen by research partners in Ethiopia (Gambella University) and Bangladesh (International Centre for Diarrhoeal Disease Research (ICDDRB)), and by Oxfam Monitoring Evaluation, Accountability and Learning (MEAL) team staff in DRC. Data collectors received an online training on the research tools. Staff translated the tools from English into the local languages and then had the opportunity to pilot them in a similar setting prior to use.

The data from the intervention logs were descriptively summarised. Qualitative data from IDIs and FGDs were imported into Nvivo 12 and thematically analysed by authors AJ and SW. A coding tree was developed deductively based on the research questions and topic guides. The coding tree was informed by frameworks outlining common domains of process evaluations [25], and then expanded based on emergent themes.

### Ethics and informed consent

This study received ethics approval from the London School of Hygiene and Tropical Medicine; the International Centre for Diarrhoeal Disease Research, Bangladesh; the Regional Health Bureau of Gambella, Ethiopia; and the Institutional Ethics Committee of the Research Center for the Promotion of Health, at the Higher Institute of Medical Techniques Bukavu, DRC. All humanitarians and crisis-affected populations involved in this research were informed about the purpose of the study in the local language, had the opportunity to ask questions and then provided written consent.

## Results

### Research participants

Interviews were conducted with a total of 8 public health engineers (PHE) and 11 public health promotion (PHP) staff who were involved in the implementation of the intervention and worked for Oxfam across the three countries. Given variations in implementation this included 9 staff in Bangladesh, 4 in DRC and 6 in Ethiopia. All staff had prior experience delivering hygiene promotion or installing and maintaining handwashing facilities in the camps where they worked. All staff had worked for Oxfam for at least a year, with some staff having been in the WASH sector for as long as 10 years.

Additionally, 151 people were involved in the FGDs across the three countries. In all three study sites a greater proportion of men participated in the research than women. Durations of displacement varied by setting, with those in Ethiopia predominantly being displaced in the last 6 months while in DRC and Bangladesh most participants had been displaced for several years. Education levels among the study population were low, with the majority of participants having primary school or no formal education. Household sizes were large across all sites but were highest in Ethiopia. Most households included children under the age of 5 or older people who are likely to be more vulnerable to diarrhoeal diseases or COVID-19 respectively. Table 2 describes the characteristics of the participants across the three settings.

**Table 2:** Summary of the socio-demographic characteristics of the crisis-affected populations who participated in FGDs

	Ethiopia (n = 48)	DRC (n = 48)	Bangladesh (n = 55)
Sex			
Male	30 (62%)	27 (56%)	33 (60%)
Female	18 (38%)	21 (44%)	22 (40%)
Age—average (range)	40 (18–75)	44 (22–70)	42.6 (18–75)
Level of education			
No formal schooling	26 (54%)	6 (12%)	41 (74%)
Primary education (partial or completed)	11 (23%)	20 (42%)	13 (24%)
Secondary education (partial or completed)	10 (21%)	22 (46%)	0 (0%)
Tertiary education (partial or completed)	1 (2%)	0 (0%)	1 (2%)
Number of people in the household—average (range)	8.25 (4–12)	6.3 (3–10)	5.4 (3–12)
Vulnerable individuals in the households			
Households with children under 5 years of age	35 (73%)	38 (79%)	37 (67%)
Households with people over 60 years of age	18 (38%)	19 (39%)	14 (25%)
Duration of living in the camp	6.3 months (3–7 months)	3.8 years (1–20 years)	4.02 years (4–5 years)
Religion			
Muslim	0 (0%)	0 (0%)	55 (100%)
Christian	40 (83%)	47 (98%)	0 (0%)
Traditional religion	7 (15%)	0 (0%)	0 (0%)
No religion	1 (2%)	1 (2%)	0 (0%)

### Description of intervention implementation based on intervention log-books

Once the OHSs were sent to the respective countries, they took an average of 129 days (range 42–278 days) to clear customs and arrive at the research sites. These delays were due to issues relating to tax legislation, permission letters and work interruptions associated with the pandemic. In Bangladesh the facilities arrived in good condition, however, in DRC and Ethiopia some items were damaged or stolen and needed to be remade or replaced locally. Delivery of MMH was also delayed in all three sites due to issues with procurement and delivery of the information, education and communication (IEC) materials for the intervention.

According to the intervention log-books there were variations in the way that the OHS and MMH intervention was implemented across the three sites. This was due to differences in the way that the interventions were planned (e.g. number of staff trained on each component and the duration of their training) and variations because of contextual priorities and the physical layout of spaces. In Bangladesh and Ethiopia, the majority of the OHS facilities were sited at household or shared latrines, while in DRC the majority were in public spaces. Accordingly, the number of people expected to use each was highest in DRC (average of 224 people per facility as compared to an average of 17 in Ethiopia and 52 in Bangladesh). In all countries people from the affected communities were consulted on the location of facilities, the height of facilities, and informed about the operation and maintenance requirements (e.g. cleaning and replenishing soap and water). At almost all facilities community members were given a supply of soap detergent and padlocks to maintain the facility (provided with the OHS kit). However, other recommended parts of the intervention were less regularly implemented, such as digging soak away pits and installing footprint cues which were designed to trigger behaviour at key times.

The MMH intervention reached 3863 caregivers across all the sites. The contextual adaption of MMH meant that a slightly different combination of activities were delivered in each of the three sites (See Supplementary Materials 2 for full details), and there were also variations in the frequency of meetings with the target population (‘dose’). A descriptive summary of the intervention components, drawn from intervention log-books is provided for each country in Table 3.

**Table 3:** Descriptive summary of the intervention in each of the countries based on the intervention log-books completed by Oxfam staff

Intervention components	Ethiopia	DRC	Bangladesh
Target population size^a^	2000 households (10,000 individuals)	1000 households (5000 individuals)	5000 households (25,000 individuals)
Duration of training provided to implementation staff about the OHS	1 h	1 day	1 day
Number of staff trained on the OHS	2	35	85
Number of OHS facilities	509	511	948
Location of OHS			
Public spaces (markets, water points, communal spaces)	62 (12%)	287 (56%)	105 (11%)
Outside a household or shared latrine	394 (77%)	143 (28%)	757 (78%)
Institutional settings (e.g. situated outside a health centre, school, youth centre, religious building, distribution site or organisation offices)	44 (9%)	16 (3%)	86 (9%)
Unspecified	9 (2%)	64 (13%)	0 (0%)
Duration of installation—average (range)	22 min (5–44 min)	55 min (30–60 min)	30 min (15–40 min)
Anticipated number of OHS users per facility—average (range)	17 (1—700)	224 (55 -1928)	52 (5—165)
Post installation steps			
Height was adjusted	154 (30%)	511 (100%)	848 (89%)
Soak away pit dug	172 (34%)	511 (100%)	226 (24%)
Footsteps to the facility installed	26 (5%)	174 (34%)	6 (0.6%)
IEC materials placed on the OHS	475 (93%)	511 (100%)	942 (99%)
Padlocks provided	509 (100%)	497 (97%)	948 (100%)
Soap (detergent) provided	509 (100%)	499 (97%)	948 (100%)
Number of facilities where all the above steps were complete	0 (0%)	172 (34%)	0 (0%)
Duration of training provided to implementation staff on MMH	2 h	2.5 h	5 h
Number of implementation staff trained on MMH	7	5	2
Number of community volunteers trained on MMH	102	20	11
Total number of people involved in MMH activities (attending at least one session)	951	2578	334
Total number of MMH activities delivered in each country (See Additional file 2 for more details)	10	9	12

^a^The number of people Oxfam intended to reach as part of their programming (based on their initial project proposals)

### Training

Training on the OHS and MMH was done online and in English for senior staff. It involved a PowerPoint presentation, and the sharing of written guides and visuals. This process was then replicated in-person for field implementation teams by those who had attended the online training. Generally, participants in the online training felt that the training on the OHS component was clearer to understand and apply than the MMH component. Participants explained that this was because if you had a basic WASH engineering background, there was nothing particularly complex about installing the OHS:

“I have received a very basic training, and it is enough for me as I am a very technical person. As an engineer it was easy for me understand what I have to do [with the OHS], as it is easy to assemble the parts in the location, so it [the training] was sufficient even though it was only 20-30 mins, it was enough.”—PHE in Bangladesh

Since the OHS and MMH were new approaches in all three countries, many participants felt that one online training was insufficient to prepare them for implementation. Instead, they recommended that it would be useful to have follow up sessions after implementation had started so that the teams could share their challenges and work towards improving the quality of the programming. This was considered useful because the training was relatively generic, but the challenges that arose during implementation often related to adapting the interventions to the context.

Staff found the complementary written guides and visuals useful to aid learning but felt that videos on the implementation of the OHS and MMH would further aid understanding and support them in training others. Staff in Bangladesh and DRC felt that delivery of the online training in English created barriers to understanding, while intermittent internet connection made it hard for staff in Ethiopia to follow the full duration of the training. Several staff across the three countries were unable to attend the initial online training resulting in them having to learn from colleagues during the implementation itself.

### Reach and accessibility

Both interventions struggled to comprehensively reach all people within the targeted settings. Staff in all countries felt that the optimal distribution of OHS facilities would be so that all households or blocks had a facility, but the amount procured meant that this was not always possible:

“More stands [OHS] are needed, for half of the community did not get handwashing facilities in the camp, there is a big shortage still.”—PHE Ethiopia

This sentiment was echoed by populations in DRC and Ethiopia who said that they would prefer to have the OHS located near household toilets rather than in shared public spaces.

In relation to MMH, the barriers to reach were related to the small-group delivery modality and the need to avoid large group gatherings during the pandemic. While those participating in the programme were exposed to all the necessary sessions, many felt that the reach was insufficient to realise behaviour change at a camp level:

“We selected 60 groups of 10 mothers per group which is 600 hundred in total but the total population [of the camp] is around 21,000. So, you wonder does this programme represent the whole camp, is it enough to create change?”—PHE Bangladesh

The OHS was perceived to be able to be used by most people in the camps. Barriers were noted for very young children due to the height of facilities and difficulties in pushing the tap up. Staff felt that the facilities could be used by people with disabilities, but these individuals may need support from others to guide them to the facilities and initially show them how to use it. Disabled people themselves reported that the OHS was easier to use than other handwashing facility designs available in the camps, but they were located too far from their homes, so in practice they rarely used them. While the interventions in Ethiopia and DRC were targeted at displaced populations, staff felt that future hygiene interventions should include neighbouring host populations who also face similar challenges (as was done in Bangladesh). During MMH implementation, staff were encouraged to actively involve men, despite mothers being the primary focus of the narrative component of the intervention. This was done across all settings, yet some staff felt the inclusion of men could be strengthened:

“The rate of participation of men compared to women was still too minimal. We focused and talked about the magic hands of mothers, and so often the men tended to stay away and it was only as the days went by that they started to integrate gradually.”—PHP DRC

### Feasibility

Staff felt that the combination of a ‘software’ and ‘hardware’ intervention made the overall implementation feasible and appropriate in all three settings as it addressed access and behavioural barriers. In the case of the OHS, feasibility was enhanced because all materials for construction were provided, the construction was considered easy to do, and the consultations with communities at the point of construction engendered greater buy-in and supported OHS maintenance. The MMH intervention was considered feasible because there was a fully designed set of activities and communication materials. However, the intervention time period was considered to be too short, particularly for the MMH component, which was time-intensive to implement:

“The time for the implementation was too short and yet if we had taken enough time we would have led … a large part of the community to change their behaviour through this approach of MMH.”—PHP DRC

“It’s an issue of behaviour change, 6 weeks is not enough time for behaviour change. There should have a continuation. After 6 weeks, then what?”—PHP Bangladesh.

### Acceptability

Implementing staff across all settings felt that the interventions were well received and that the acceptability of both MMH and the OHS improved over time. Characteristics of the OHS that staff felt improved its acceptability were that it minimised the amount of water used for handwashing, had an innovative and attractive design, included a mirror and footprints to nudge behaviour, and that it served many people before the soap and water needed replacing. Staff estimated that about 200 people could wash their hands at the facility before the soap and water needed to be replaced, meaning that it could sometimes be 2–3 days between soap and water refills.

The majority of FGD participants, across all countries, were generally positive about the design of the OHS as well. Participants felt that the colours of the OHS, the mirror, and the unique modern look of the facility made it desirable to use. In terms of its functionality, participants like that it had both soap and water dispensers, that more than one person could use it at the same time, that it had a pipe to facilitate good drainage, that it was water saving and that the tripod design made it relatively stable when in use.

However, challenges with the design were also identified by staff and populations alike. One initial challenge was that the OHS was not immediately recognised to be a handwashing facility because of its novel design and was therefore sometimes treated with suspicion:

“They [populations] were not aware of what it was about when we brought the kits to the camps. It was only after several explanations that the community came to understand that it was about hand washing, but despite that, there were some people in the community who still did not accept that their family members could use it for hand washing, they said that the presence of the mirror was actually a camera and worried that they could be followed by anyone.”—PHP Bangladesh

“When I first saw this, I was amazed by the beautifulness of it and the mirror, but I didn’t know what it was!”—Male FGD participant in Ethiopia

“At the beginning, people were fearing the handwashing facility because of its form and features, they were confused by the mirror and though it might have been a camera and the tripod they thought that was similar to what was used for a gun.”—Male FGD participant in DRC

Staff and populations in all three settings also agreed that the width of the legs of the facilities created challenges given that the OHS facilities often had to be installed in small, congested spaces:

“Another issue is space, the legs are spread out, that’s why it needs so much space, but in the camp space is a major constraint to install the station, I think.”—PHP Bangladesh

In all three countries people did not like the mode of dispensing water (which requires users to push a thin metal nozzle up to release water). The following discussion among male FGD participants in DRC summarises some of the issues people had with the OHS tap:

“Interviewers: What do you not like about the OHS?

Participant 1: The way it dispenses water with the metal tap and when you are washing your hands you can feel a little bit like it is grating against your skin….

Participant 2: [Handwashing facilities which have] the foot pusher are great, because they told us that when you touch things like this tap on the OHS you can bring microbes onto your hands. So we are asking to change it [the OHS] to have a foot pusher….

Participant 3: You always come and hear us and sometime you don’t come back with an answer…So we want to be clear we are asking Oxfam to modify or change the OHS

Interviewer: Does that mean that you don’t like the OHS?

Participant 3: We like it, but only not this metal tap”

Staff also felt that this water dispensing mechanism was unfamiliar and uncomfortable and that the flow rate was insufficient to easily facilitate handwashing:

“I also, like the community, did not like the use of the tap; behind the palm of the hand hurt every time the tap was used, I found in the long run that if you wash 2, 3 times like that, it can always leave lesions and that's what I didn't appreciate.”—PHP DRC

“The flow rates is very low, so people have no patience to wait for a long time to wash their hands.”—PHE Ethiopia

In Bangladesh and DRC, staff felt that refilling the OHS water containers was still inconvenient for populations due to distances from water points, although this was not raised by populations themselves. Staff therefore emphasised that provision of the OHS did not go far enough to address the broader issue of access to convenient water sources:

“The problem still is that the water source is not coming from a convenient place. Suppose, the station is beside the latrine but then the water source might be 25–30 feet away… As it’s a hilly area, to fetch water from this distance is troublesome work for them. In that case, the main hindrance of the use of the OHS and the practice of hand washing is the availability of convenient water sources.”—PHP Bangladesh

In relation to MMH, staff reported that populations thought the activities were surprising, inspiring and different from usual health education sessions:

“Here we are giving hygiene message by telling stories, providing materials, with activities etc. It is far different from other hygiene message delivery systems.”—PHP Bangladesh

However, encouraging attendance at the small group sessions was challenging initially because women often had to make childcare arrangements or delay other daily tasks, and men often had to give up money earning activities to attend:

“People struggled to participate in the regular MMH sessions because of their busy lives and competing priorities. At the beginning it was not easy…but they ended up understanding the importance, they ended up starting to participate without being forced.”—PHP DRC

Staff often explained the importance of the sessions by reiterating the modes of COVID-19 transmission. Given that populations were worried about COVID-19, this helped present the sessions as relevant and important.

Participants reported that they enjoyed sharing the MMH story with their children, that the illustrations were attractive, that they valued being able to take some of the materials home, and that they were intrigued by some of the demonstrations (such as the pepper and soap activity). However, many of the FGD participants had not been directly involved in the MMH programme, particularly in Bangladesh. Community members felt that the main difference between MMH and other hygiene promotion programmes was the strong focus on family roles and caregiving responsibilities.

### Perceived ownership

Some challenges were reported by staff in terms of building a sense of community ownership and shared responsibility for managing the OHS:

“A common challenge in the community was that…we failed to make them understand that it is now ‘our’ property and it is not ‘my’ property. And so they are all responsible and not solely one person.”—PHE Bangladesh

This seemed to improve through community dialogue and by dividing up responsibilities for replenishing the soap and water.

Most FGD participants did report that they played a role in replenishing the soap and water at the OHS indicating that this responsibility was being shared and was undertaken by people of different ages and genders that lived nearby the facility. People reported it was relatively easy to drain any remain water or soap, clean the tanks and replenish the soap and water. However, people with disabilities said they were unable to refill and clean the OHS independently because of the weight and height of the containers. On average people reported refilling the OHS once per day, but this ranged from 5 times per day if the OHS was located in busy public areas, to every 2 days if it was only shared between a small number of families. Across all the settings, participants viewed the OHS as being owned by the camp residents:

“Yes, I do feel that its mine. Everyone likes it and uses it as if it’s their own property.”—FGD with adult women in Bangladesh

Those who contributed to refilling the OHS were typically seen as primary ‘owners’ of the stations by others.

### Durability and sustainability of the OHS

The OHS facilities were seen by staff as being much more durable than other pre-existing types of handwashing facilities in the camp (these included tippy-taps [26] or buckets with taps) and that it would last up to two years even with high volumes of use. Populations gave similar estimates for how long they thought the OHS would last, ranging from six months to three years. The high quality of the facility also meant that populations were more concerned about it being damaged, stolen or misused and therefore people often wanted to bring it indoors at night. This created its own challenges given that the legs are too wide to fit through most doorways, and if the handwashing facility is moved inside, it is not always available for others to use when they need it. In Ethiopia, staff reported having to make repairs to the OHS facilities on a weekly basis because children were often playing with the facilities or because people did not know how to use the taps and therefore broke them. Reported challenges were that the soapy water tank started to leak or that populations did not correctly mix the soapy water, which caused blockages in the tap. Staff in DRC also mentioned that if the OHS is located in the sun for most of the day the plastic starts to get damaged and weak, however this may have been more of a perception than a reality given that the facility is made from UV resistant plastic. Staff raised some concerns about long-term maintenance of the facilities given that all parts were imported and not locally available.

### Reported handwashing behaviour and use of the OHS

Prior to the implementation of the interventions, FGD participants reported that handwashing was generally easy for most people to practice but that children, older people and people with disabilities often needed support to wash their hands. Across all three countries there was a high level of understanding about why handwashing was important and the critical times when hands should be washed:

“I wash my hands with soap at six important times in a day…. Before cooking, before eating, before feeding others, before toilet, after cleaning children’s defecation, after coughing, or sneezing.”—Female FGD participant in Bangladesh

“The reasons we wash hands is to prevent diseases such as cholera, Ebola, COVID, and diarrhoea and it’s because we are using our hands to touch everything so they can get contaminated.”—Male FGD participant in DRC

Water and soap shortages were cited as a common challenge preventing handwashing across the settings. Some participants also reported that it was harder to practice handwashing when outside the home, during the rainy season, or at certain times of the day (e.g. after dark or before eating). Across all settings, economic hardships and hunger were reported to be things that could easily interrupt good hygiene practices.

FGD participants reported that during the pandemic they had increased the frequency and duration of their handwashing due to concerns about the transmission of the virus. Repeated hygiene promotion sessions (MMH and sessions run by other organisations) helped reinforce the importance of handwashing during this time. FGD participants typically reported that the OHS made it easier to wash hands because there were more facilities closer to people’s homes and in key public settings, therefore the facilities themselves acted as a reminder for handwashing at critical COVID-19 prevention moments. Participants also said that the design of the facilities made it more desirable to practice handwashing and meant that it was easier to ensure that soap was always available. In Bangladesh, FGD participants generally considered the OHS to be the most commonly available handwashing facility in the camp, enabling regular handwashing practice. In Ethiopia, participants reported that the OHS was used less frequently than other facilities as it is one of the least common handwashing facilities. Participants in Ethiopia felt that a lack of facilities prevented them from washing their hands as frequently as they would have liked:

“I am using mobile objects like jugs [to facilitate handwashing] because the facilities provided by Oxfam were not distributed enough to everyone, due to this we use jug whenever we want to wash our hands at home.”– Female FGD participant in Ethiopia

In DRC, few other handwashing facilities existed, so while the OHS was not found everywhere it was still commonly used.

## Discussion

This qualitative assessment provides an indication of the feasibility and acceptability of a handwashing promotion package that combines both ‘hardware’ and ‘software’ components, and which was delivered at scale during the COVID-19 pandemic among highly vulnerable, displaced populations. This intervention filled a number of gaps that have previously been identified in hygiene promotion in humanitarian emergencies, such as the inclusion of a theory-informed behaviour change component alongside handwashing hardware distributions, handwashing promotion messages that were not solely health based, and accompanying research to evaluate the acceptability and use of hardware provided in interventions [27].

Our findings were relatively consistent across contexts, and there was a high level of agreement between the perspectives of intervention implementers and the perspectives of crisis-affected populations. Overall, the combined OHS and MMH intervention was viewed as novel and appealing by implementing staff and crisis-affected populations. In both cases this appeared to be because greater effort had been put into the design of both intervention components. In the case of the OHS, it was seen as being not just a functional facility but also one that was attractive and cued behaviour. In the case of MMH, the strong focus on motives, storytelling and creative activities and materials helped it stand out from standard health-centric hygiene education programmes. The feasibility of the interventions may have been facilitated by the fact that staff had prior experience in health promotion and WASH engineering making both interventions relatively easy to implement. Strengthening these capacities may be needed if implemented by other actors. The acceptability of both interventions was also facilitated by high levels of handwashing knowledge prior to the intervention and concern about the COVID-19 pandemic.

However, our findings also highlighted that even when an intervention has been well-designed, there are many contextual aspects that need to be considered, and unintended consequences which can affect the acceptability of an intervention. In our study we identified that the OHS could be improved through redesigning the tap and the space consumed by the structure’s legs. While the OHS was designed to be a shared facility, participants expressed a high demand for durable handwashing solutions that can be kept at the household level and this could merit future research and development given that current household designs have limited durability and scale [28]. In all three countries substantial challenges were faced in importing the OHS into the country. Customs delays and import challenges are not unique to this project and have been recognised to hamper humanitarian aid more broadly [29, 30]. However, these challenges would need to be systematically addressed if these interventions are to be deployed in the future during acute crises. Staff also raised concerns about the long-term maintenance of the OHS given that parts were not locally available. Localisation of humanitarian supply chains has been noted to have positive effects on local economies, aiding recovery from crises and contributing to sustainability [31, 32]. However, logistics and supply chains have historically been under-researched within the hygiene sector and therefore this topic merits further work. Given that some of the missing OHS parts were subsequently procured locally in Ethiopia, this may create opportunities to increase the localisation of supply chains for the product in the future. Our findings indicated that the sustainability and use of the handwashing facilities in crisis-affected settings may also need to factor in water scarcity, intermittency and access, something that is becoming a growing challenge in crisis-affected regions in recent years [33, 34].

Issues with the intuitiveness of the OHS design and willingness to participate in MMH were ultimately overcome through more effective community engagement. Future use of the combined interventions could more effectively build this into programming and make sure to address misconceptions from the start. Similar challenges related to intuitiveness and product trust have been encountered in the design and roll-out of other novel emergency handwashing products [19]. The implementation of the MMH intervention varied substantially by country making it challenging to compare. In part this was due to the context adaptation made by each country team, however in future implementation this could be accounted for by strengthening the training and ongoing support to implementation staff. Future process evaluations of MMH or other hygiene promotion programmes could benefit from observing the intervention at each stage and speaking with implementation staff to better understand how contextual adaptations are made.

**Fig. 3:**
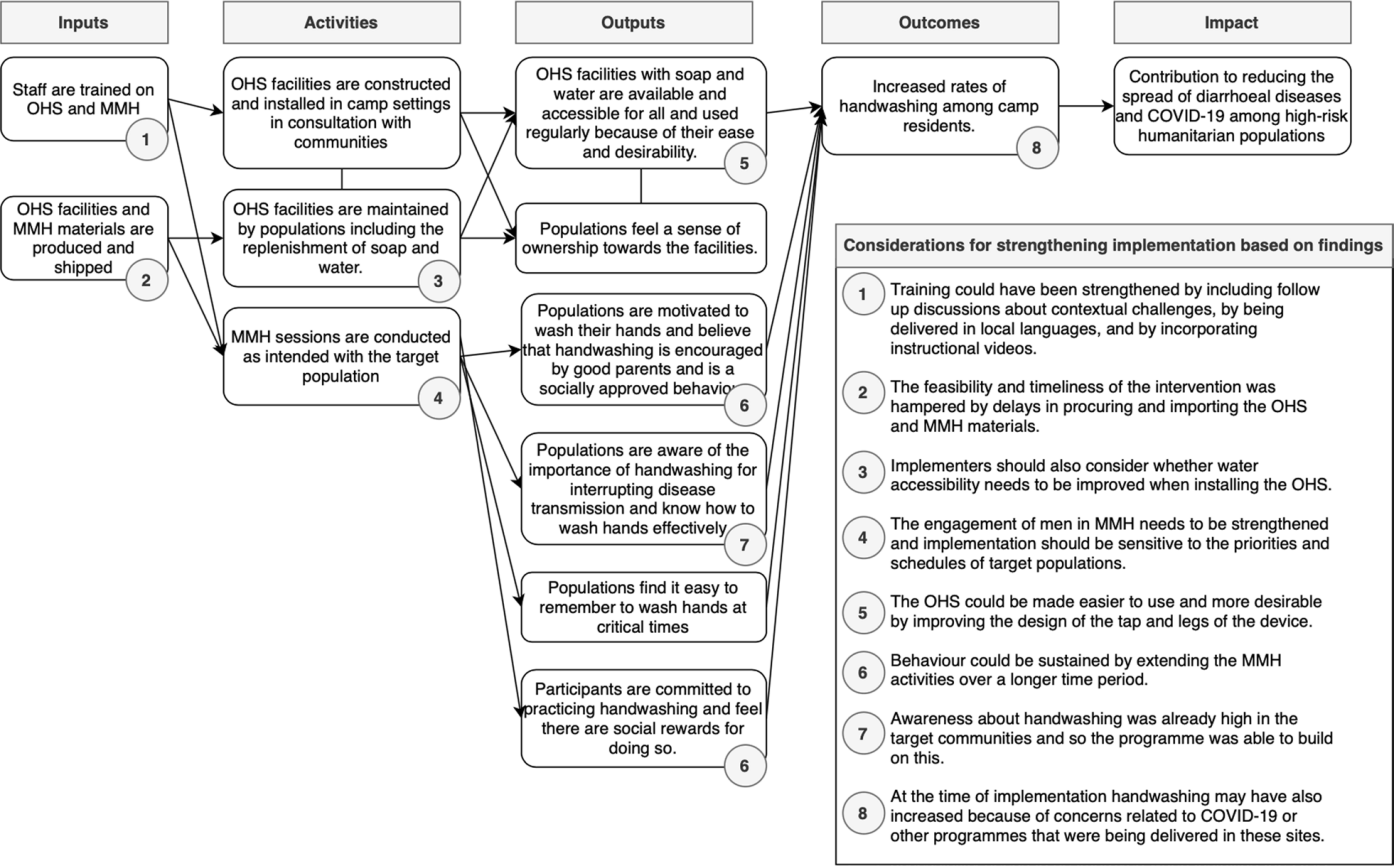
Adapted theory of change showing how the combined use of the Oxfam Handwashing Stand and Mum's Magic Hands could be strengthened when implemented in the future

Despite the many appealing aspects of the OHS and MMH interventions, the implementation challenges outlined above are likely to have prevented the programme from fully and sustainably achieving its intended outcome of improving handwashing rates within the camps. However, in Fig. 3 we provide an adapted theory of change which describes considerations for future use of these combined interventions. If these challenges could effectively be overcome, then a combination of interventions like these is likely to be a viable model for improving handwashing behaviours in camp settings.

### Limitations

This research was a collaboration between research institutions and operational partners amid the challenging circumstances of the pandemic. This necessitated that much of the training on data collection methods and processes for quality monitoring had to be done remotely and across time zones and language barriers. This approach had the benefit of engaging camp residents and operational staff in the data collection and strengthening local research capacities. However, it also introduced potential data inaccuracies and biases. For example, the intervention delivery and data collection were substantially delayed under this project, which led to compromises with the quality of the translation and transcription of interviews and FGDs. Therefore, it is likely that some data from the FGDs in Ethiopia is missing and much of the FGD data from DRC may be an over-simplification of the nuanced reflections of populations. Given that transcription can have a huge bearing on the quality of qualitative data analysis and interpretation [35, 36] we would encourage future researchers to consider how delays may affect this aspect of their work. Additionally, the involvement of hygiene monitoring staff within all the camps and Oxfam staff in DRC (due to the absence of an academic institutional partner in this setting) may have increased social desirability within participant responses, causing people to be more likely to report positive opinions of the interventions. This may especially have been an issue for the IDIs conducted by Oxfam staff in DRC.

FGD participants were selected based on their familiarity and use of the OHS facility. However, exposure to MMH did not necessarily correlate with use of the OHS. Therefore, FGD discussions were unable to generate rich data on MMH implementation given that many participants had not been exposed to that part of the intervention at all. Future implementation might explore opportunities to align the target populations more closely for these two interventions.

Our study only described reported and perceived changes to handwashing practice. Self-reported handwashing behaviour is known to often overestimate actual handwashing practices [12]. Data on observed use of the handwashing facilities was collected in these sites and will be reported separately (manuscript under development). Many of the other constructs that were of interest to this study, such as ‘sense of ownership’ are hard to measure and were explored only superficially in this study. The importance of ownership for handwashing facility maintenance would benefit from further research which employs validated scales around concepts such as psychological ownership [37].

## Conclusion

Historically hygiene infrastructure and hygiene promotion have been viewed as different types of programming, the former being the remit of engineers and the latter being the remit of public health staff aiming to change behaviour. Together with prior research [38–42], this study supports the fact infrastructure and products should be viewed as a core enabler of handwashing behaviour and a necessary complement to delivering acceptable hygiene programming in humanitarian contexts. While our study focused only on evaluating the OHS and MMH intervention components, we recognise that many of these challenges are not necessarily unique to these interventions and are therefore worthy of consideration for the implementation of other hygiene programmes in humanitarian situations and outbreaks. Our findings support need for creative and theory driven intervention design, contextual adaptation, and adaption or re-design based on the experiences of implementers and crisis-affected populations.

## Supplementary Information

**Additional file 1.** Existing Handwashing facility Types

**Additional file 2.** Detailed description of MMH

## Data Availability

The datasets used and/or analysed during the current study are available from the corresponding author on reasonable request.
