# Supplementary material for: Facilitating hand hygiene in displacement camps during the COVID-19 pandemic: a qualitative assessment of a novel handwashing stand and hygiene promotion package
Source: Confl Health. 2022 Dec 16;16:65. doi: 10.1186/s13031-022-00492-8 (PMC9756724; doi:10.1186/s13031-022-00492-8)

# Supplementary Material 1:

Images of existing handwashing facilities that were present in the study sites prior to intervention implementation.

**Bangladesh:**


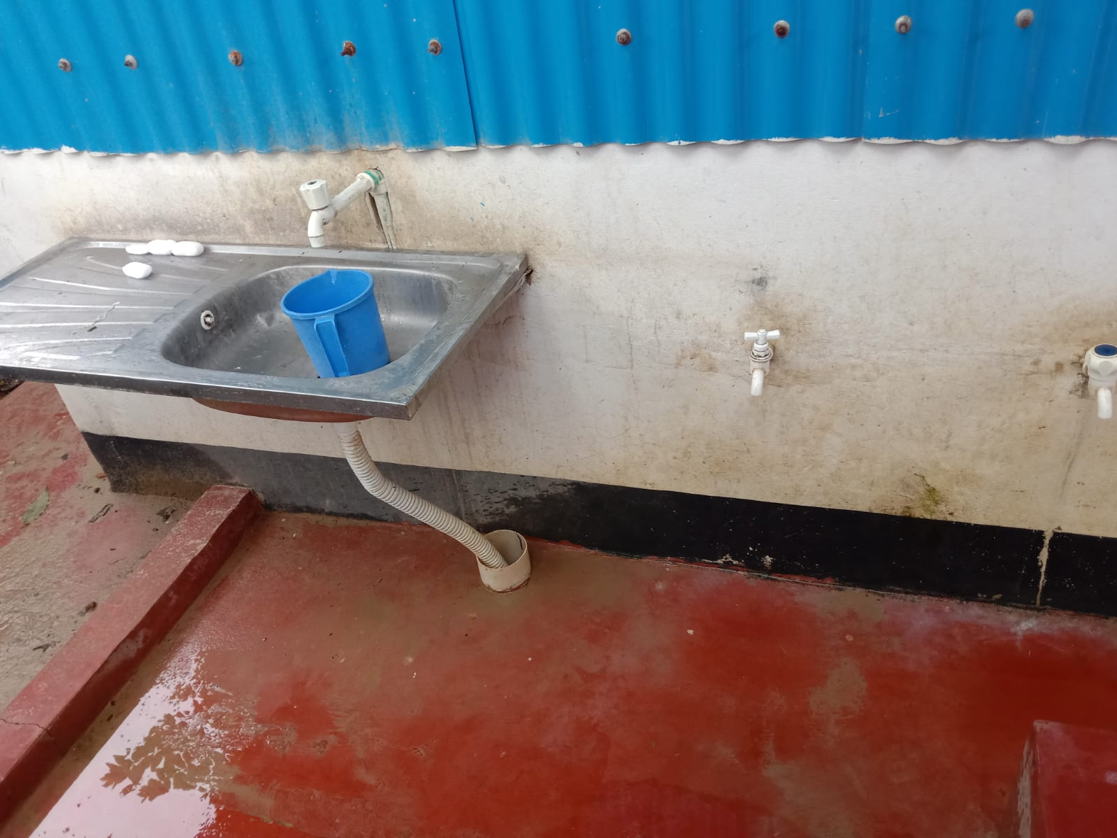

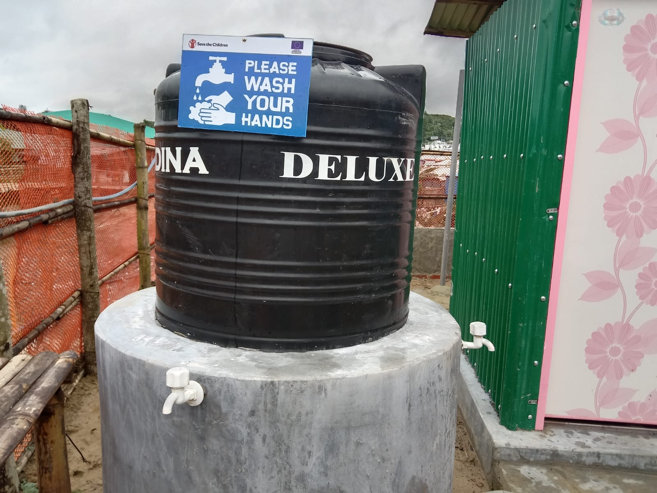

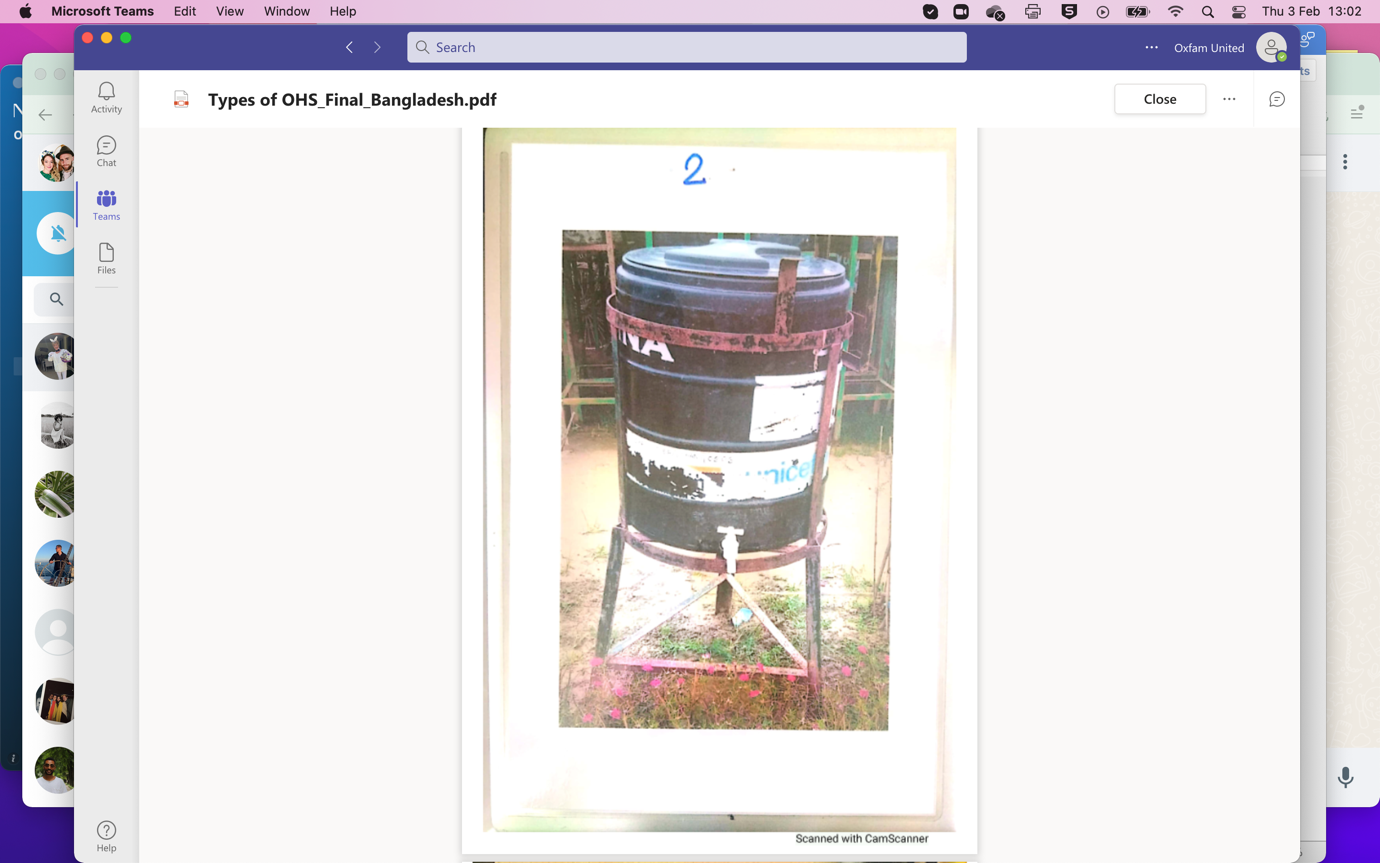

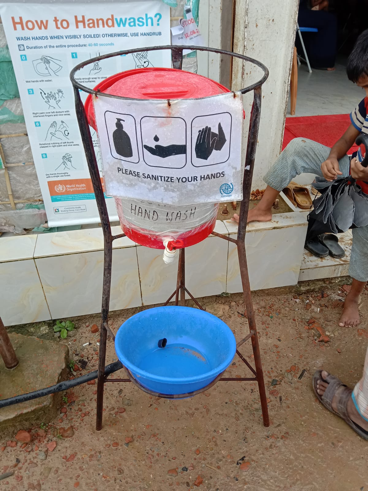

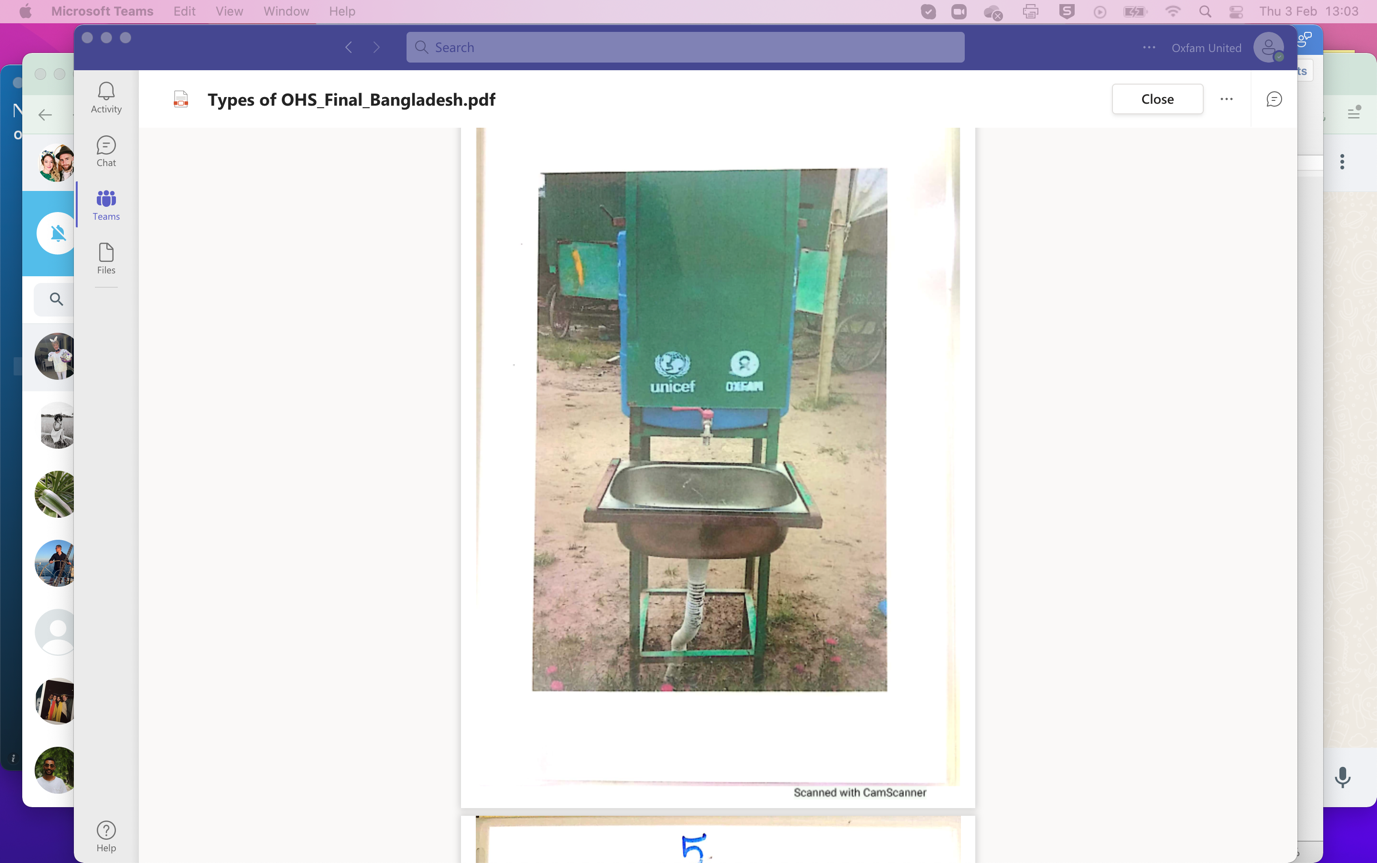


**Ethiopia:**


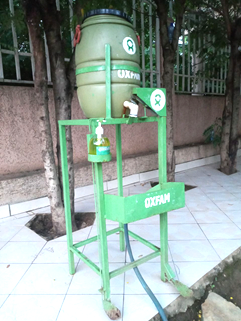


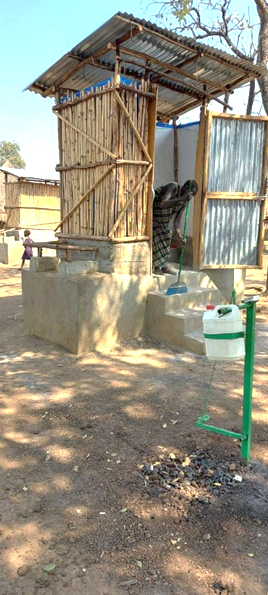


**DRC:**

**DRC:**


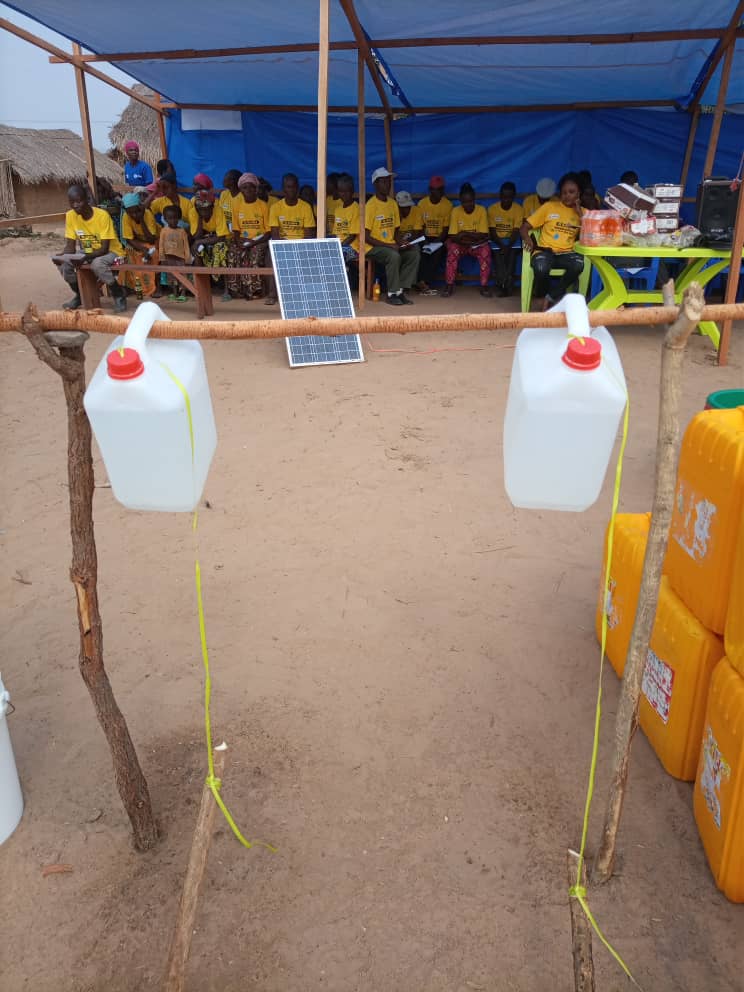

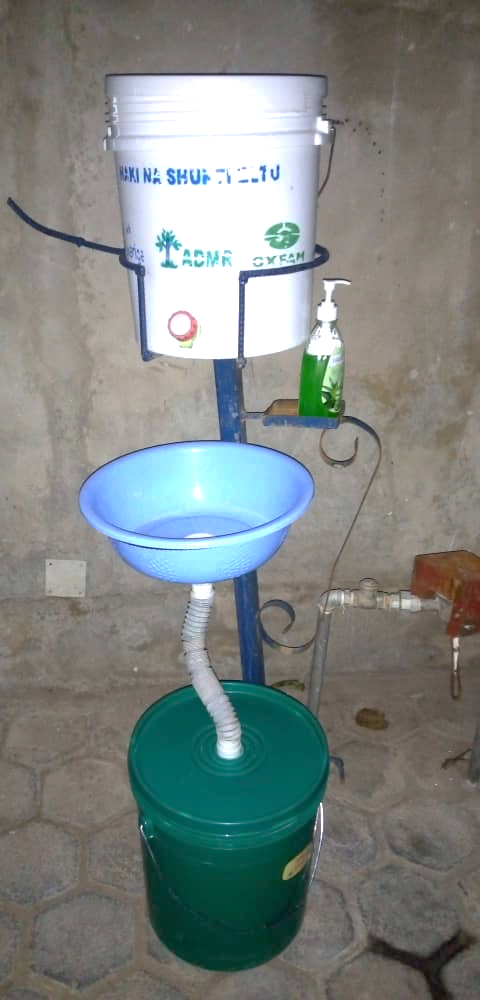

Supplement: Supplementary file 1 — Additional file 1. Existing Handwashing facility Types [file 13031_2022_492_MOESM1_ESM.docx]
