# Supplementary material for: Facilitating hand hygiene in displacement camps during the COVID-19 pandemic: a qualitative assessment of a novel handwashing stand and hygiene promotion package
Source: Confl Health. 2022 Dec 16;16:65. doi: 10.1186/s13031-022-00492-8 (PMC9756724; doi:10.1186/s13031-022-00492-8)
Supplement: Supplementary file 2 — Additional file 2. Detailed description of MMH [file 13031_2022_492_MOESM2_ESM.docx]

# Supplementary Material 1

table 1: a description of each component of mum’s magic hands and the intended purpose

| **Type of activity** | **Description of MMH component** | **Purpose or desired effect on behaviour** | **DRC** | **Ethiopia** | **Bangladesh** |
| --- | --- | --- | --- | --- | --- |
| Infrastructural change | Footprints are added on floor leading up to the OHS handwashing facility. | Footsteps are designed to make handwashing hard to avoid by cuing behaviour following certain courses of action (such as using the toilet). | ✓ | ✓ | ✓ |
| Small group session | An overview of the importance of handwashing and reminders about how and when to wash hands. The OHS facility is used to demonstrate this. | Improve knowledge about how to wash hands and critical times for handwashing. Introduce people to the OHS facility. | ✓ | ✓ | ✓ |
| Small group session | Reading of the MMH story about a mother who encourages her children to wash their hands so they can be successful in life. Discussion around what the story means and whether participants can relate to it. | The story is designed to motivate participants to wash hands by associating the behaviour with nurturing (being a good parent). | ✓ | ✓ | ✓ |
| Small group session | A handwashing demonstration is done using the OHS facility. This involves the community volunteer dipping their hand in coloured powder and then washing their hands, initially with just water and then with soap. | The coloured powder allows the participants to comment on how well the handwashing has been done and notice the difference that soap makes to removing it from hands. | ✓ | ✓ | ✓ |
| Small group session | A voting exercise is conducted where people are asked to vote according to whether they notice other people always washing their hands at critical times. The results of the vote are discussed, and it is mentioned that if some people do not regularly wash their hands it could affect the health of everyone in their community. | This is designed to draw attention to community norms around handwashing and make people aware that others notice their handwashing behaviour. |  | ✓ | ✓ |
| Small group session | A container of water is brought out and some pepper is added to the surface. A participant is asked to dip their finger in the water – the powder remains as it is. The same participant is then asked to put soap on their finger and re-dip their finger into the water. This time the powder moves away from their finger suddenly. | This is designed to surprise participants and make them realise how important soap is in making hands clean. |  | ✓ | ✓ |
| Small group session | Participants are encouraged to develop a bedtime story related to handwashing. This can build on the Mum’s Magic Hands story or be their own. | This is designed to associate handwashing with caring for your child and encouraging them to wash their hands. By telling stories at night to their children it is hoped this acts as a reminder to parents and children alike. |  | ✓ | ✓ |
| Small group session | Development of an action plan for how the OHS facility will be maintained, and the soap and water regularly replenished. | This aims to encouraged shared ownership of the facility and get people to express their intentions to maintain it. | ✓ |  | ✓ |
| Materials | Scratch cards are given out to mothers to help them encourage their children to wash hands with soap. The scratch cards have 21 squares with pictures of the MMH storyboard, and children get to see each card after washing hands. | The scratch cards are designed to reward children for good handwashing practice and get them to associate handwashing with the story of Mum’s Magic Hands. | ✓ | ✓ | ✓ |
| Materials | Posters are given to participants, and they are encouraged to put these up in their homes as reminders. | The posters designed to remind people about effective handwashing technique and key handwashing moments | ✓ | ✓ | ✓ |
| Materials | Participants are given stickers to add to the doors of their latrines or elsewhere in their homes. | The stickers are designed to remind participants to wash their hands at the key moments. | ✓ | ✓ | ✓ |
| Materials | Participants who have attended all of the sessions receive a certificate. | The certificates are given to mothers to recognise what they have learned and practiced and to indicate that they are now a ‘Magic Mum’ |  |  | ✓ |
| Large group session | Local theatre performance enacting the Mum’s Magic Hands story and demonstrating key moments for handwashing | Helped to bring the story to life and share the story with a broader group of the community. Help reinforce key moments for handwashing. | ✓ |  |  |

Notes:

- The small group sessions typically brought together 10-30 neighbouring individuals and were generally disaggregated by gender and age.
- The activities conducted in small group sessions were combined in different ways in each of the countries. For example, in Bangladesh groups met on four occasions, at 15-day intervals. In Ethiopia the groups took part in two Mum’s Magic Hands sessions in order to be able to go through all the activities, with each session lasting about 45 minutes. In DRC the activities were combined into one session with each group of individuals.
- In DRC the activities were delivered alongside other ongoing WASH related activities.
